# Meteorological Table for the Month of March, 1852

**Published:** 1852-05

**Authors:** Lawrence Young


### METEOROLOGICAL TABLE,

FOR THE MONTH OF MARCH, 1852.

BY LAWRENCE YOUNG, ESQ.

| Day of month, .... | Morning, ........ | 2 o'clock, P. M., .... | Evening, ........ | Mean temperature, .. | Barometer, ........ | Rain-guage, ........ | Course of wind, ...... | Remarks. |
|---|---|---|---|---|---|---|---|---|
| 1 | 35 | 68 | 63 | 55 | 29.07 | | e. | Variable. |
| 2 | 32 | 42 | 35 | 36 | 29.22 | | n. n. w. | Cloudy. |
| 3 | 26 | 47 | 45 | 39 | 29.58 | | e. | Clear. |
| 4 | 40 | 47 | 48 | 45 | 29.32 | 1.10 | e. | Cloudy. |
| 5 | 57 | 57 | 52 | 55 | 29.18 | 1.09 | calm. | Do. |
| 6 | 37 | 49 | 44 | 43 | 29.21 | 07 | n. e. | Variable. |
| 7 | 34 | 53 | 49 | 45 | 29.29 | | n. n.e. | Do. |
| 8 | 52 | 70 | 53 | 58 | 29.17 | 82 | s. s. w. | Do. |
| 9 | 64 | 48 | 45 | 52 | 28.93 | 24 | swly. | Cloudy. |
| 10 | 38 | 50 | 45 | 44 | 29.27 | | n. e. | Do. |
| 11 | 37 | 67 | 61 | 55 | 29.27 | | calm. | Variable. |
| 12 | 59 | 63 | 60 | 61 | 29.30 | | s. s. w. | Cloudy. |
| 13 | 58 | 73 | 71 | 67 | 29.14 | | s. w. | Do. |
| 14 | 63 | 69 | 60 | 64 | 28.94 | | swly. | Variable. |
| 15 | 38 | 61 | 51 | 50 | 29.10 | | calm. | Clear. |
| 16 | 43 | 70 | 60 | 58 | 29.07 | | w. | Do. |
| 17 | 37 | 31 | 27 | 32 | 28.91 | 13 | n. n. w. | Cloudy. |
| 18 | 12 | 36 | 29 | 26 | 29.21 | | w. | Clear. |
| 19 | 17 | 32 | 24 | 24 | 29.35 | | wly. | Variable. |
| 20 | 13 | 33 | 32 | 26 | 29.37 | | w. s. w. | Do. |
| 21 | 34 | 45 | 35 | 38 | 29.10 | | s. s. w. | Cloudy. |
| 22 | 24 | 49 | 43 | 39 | 29.— | | swly. | Variable. |
| 23 | 37 | 45 | 35 | 39 | 28.89 | | n. n. w. | Do. |
| 24 | 42 | 61 | 55 | 53 | 28.74 | | s. s. w. | Do. |
| 25 | 36 | 79 | 70 | 62 | 28.90 | | w. | Do. |
| 26 | 65 | 82 | 58 | 68 | 28.90 | 56 | s. w. | Clear. |
| 27 | 39 | 41 | 39 | 40 | 29.12 | | n. | Cloudy. |
| 28 | 38 | 55 | 53 | 49 | 29.16 | | e. | Do. |
| 29 | 57 | 76 | 70 | 68 | 29.09 | | s. s. w. | Variable. |
| 30 | 61 | 71 | 69 | 67 | 29.02 | | s. s. w. | Cloudy. |
| 31 | 37 | 48 | 41 | 42 | 28.92 | 67 | w. s. w. | Variable. |
| | | | | 48.4 | | 4.59 | | |

